# Targeting HIF-2α in the Tumor Microenvironment: Redefining the Role of HIF-2α for Solid Cancer Therapy

**DOI:** 10.3390/cancers14051259

**Published:** 2022-02-28

**Authors:** Leah Davis, Matthias Recktenwald, Evan Hutt, Schuyler Fuller, Madison Briggs, Arnav Goel, Nichole Daringer

**Affiliations:** 1 Department of Biomedical Engineering, Rowan University, 201 Mullica Hill Rd, Glassboro, NJ 08028, USA; davisl57@rowan.edu (L.D.); reckte75@rowan.edu (M.R.); hutte8@rowan.edu (E.H.); briggs84@students.rowan.edu (M.B.); goelar84@students.rowan.edu (A.G.); 2 Department of Biomedical Engineering, Columbia University, New York, NY 10027, USA; srf2165@columbia.edu

**Keywords:** HIF-2α, HIF-1α, tumor microenvironment, tumor hypoxia, hypoxia-inducible factor

## Abstract

**Simple Summary:**

Hypoxia is defined as the inadequate supply of oxygen in tissues. Regions of acute and chronic hypoxia are a universal feature of the tumor microenvironment and a known driver of tumor progression and therapeutic resistance. As oxygen levels decrease, transcription factor HIF-1α and HIF-2α stabilize and positively regulate the hypoxic response, modulating many of the cell’s defining functions. As a result, HIF-α activation in response to tumor hypoxia can drive tumor progression, making HIF-1α and HIF-2α the primary targets for therapeutic intervention. However, although HIF-α can both sequentially and differentially regulate the hypoxic response, the role of HIF-2α is vastly under-considered. In this review, we discuss the role of HIF-2α in physiology and tumor progression and the differences between HIF-1α and HIF-2α in structure, function, and regulation of the hypoxic response. Notably, we conclude that cancer therapeutics which target HIF-2α have the potential to supplement current solid tumor fighting strategies.

**Abstract:**

Inadequate oxygen supply, or hypoxia, is characteristic of the tumor microenvironment and correlates with poor prognosis and therapeutic resistance. Hypoxia leads to the activation of the hypoxia-inducible factor (HIF) signaling pathway and stabilization of the HIF-α subunit, driving tumor progression. The homologous alpha subunits, HIF-1α and HIF-2α, are responsible for mediating the transcription of a multitude of critical proteins that control proliferation, angiogenic signaling, metastasis, and other oncogenic factors, both differentially and sequentially regulating the hypoxic response. Post-translational modifications of HIF play a central role in its behavior as a mediator of transcription, as well as the temporal transition from HIF-1α to HIF-2α that occurs in response to chronic hypoxia. While it is evident that HIF-α is highly dynamic, HIF-2α remains vastly under-considered. HIF-2α can intensify the behaviors of the most aggressive tumors by adapting the cell to oxidative stress, thereby promoting metastasis, tissue remodeling, angiogenesis, and upregulating cancer stem cell factors. The structure, function, hypoxic response, spatiotemporal dynamics, and roles in the progression and persistence of cancer of this HIF-2α molecule and its *EPAS1* gene are highlighted in this review, alongside a discussion of current therapeutics and future directions.

## 1. Introduction

Hypoxia, the inadequate supply of oxygen in tissues, is an intrinsic property of the tumor microenvironment (TME), and is present in nearly all solid cancer sites [1,2]. Tumor hypoxia leads to the activation of the hypoxia-inducible factor (HIF) signaling pathway. HIFs are α,β heterodimeric transcription factors that maintain oxygen homeostasis by mediating the expression of over 1000 genes involved in modulating many of the cell’s defining functions, including metabolic remodeling, angiogenic signaling, differentiation, and migration [3]. As a result, HIF activation in response to tumor hypoxia can drive tumor adaptation and development. In fact, hypoxia is a known poor prognosis marker, driving therapy resistance [4,5], heterogeneity [6], angiogenesis [7,8], metastasis [9], and overall tumor progression [10,11]. The complex capabilities and development of tumors, rationalized as the “hallmarks of cancer”, are all influenced by hypoxia in the TME, positioning hypoxia at the forefront of tumor progression [12,13]. Thus, exploiting the HIF pathway for therapeutic intervention is a potential strategy for treating solid cancers and is the subject of considerable current research in the fields of cellular biology and oncology.

HIFs are composed of three α-subunits (HIF-1α, HIF-2α, and HIF-3α), also known as the oxygen-sensing subunits, and three nuclear β-subunits (HIF-1β, HIF-2β, and HIF-3β). Molecular oxygen concentrations negatively regulate the stability of the α-subunit through a hydroxylation reaction which initiates the ubiquitin–proteasome degradation pathway. As cellular oxygen levels decrease, HIF-α stabilizes and translocates to the nucleus, where it can dimerize with HIF-β. Dimerization of any α-subunit with any β-subunit is sufficient to become a functional transcription factor, although individual subunits can also modulate aspects of cellular processes, including the synthesis of DNA, RNA, and proteins to maintain oxygen homeostasis [14,15]. HIF-1α and HIF-2α positively regulate the HIF response and are considered to be the primary targets for therapeutic intervention [16,17]. HIF-1α is the only α-subunit that is both constitutively transcribed and ubiquitously expressed, and therefore has been the primary focus of hypoxia research since its discovery in 1991 [18,19,20]. Namely, the structure [21], function [22], and role of HIF-1α in tumor progression [23] have been thoroughly reviewed. HIF-2α is highly homologous to HIF-1α, with 48% conserved amino acid identity, primarily in the structural and functional motifs. As a result of these similarities, HIF-1α and HIF-2α share many defining features that distinguish them as primary targets, including negative relationships with oxygen, roles as hypoxia-induced transcriptional activators, and DNA binding domains [17]. However, despite this homology, HIF-2α exhibits vastly different and distinct expression patterns, physiological roles, regulatory controls, and gene specificity in oxygen homeostasis compared to HIF-1α. Specifically, while HIF-1α is ubiquitous, HIF-2α is primarily expressed in highly vascularized organs like the heart, liver, lung, brain, kidney, intestines, pancreas, and uterus [24,25,26]. In addition, despite many overlapping downstream targets and the same DNA binding domain, HIF-1α and HIF-2α have independent binding sites, targets, and optimal oxygen concentrations, with HIF-2α mediating the chronic hypoxic response [27]. In general, HIF-1α induces genes that regulate metabolic reprogramming, vascularization, apoptosis, and nitric oxide production, while HIF-2α contributes to controlling oxidative stress, RNA transport, cell cycle progression, and vascular remodeling [28]. Recently, it was discovered that HIF-2α evolved after HIF-1α and is only found in vertebrates, while HIF-1α is phylogenetically conserved in metazoans [29]. This discovery suggests that vertebrates require additional oxygen regulation beyond what HIF-1α provides and that HIF-2α may have a more diverse role than previously believed. Therefore, it is evident that the hypoxic cellular response is dynamic, spatiotemporally regulated, and context-dependent, with differential and even sequential HIF-1α and HIF-2α activity. Similarly, the TME resembles the hypoxic response where spatiotemporal dynamics define solid tumor progression [30]. Hence, it is of no surprise that hypoxia is associated with poor prognosis. Thus, further elucidating the role of HIF-2α in both health and tumor progression may lead to novel targets and approaches to overcoming tumor hypoxia.

Tumor hypoxia leads to resistance to standard cancer therapies, specifically radiotherapy and chemotherapy [31]. Currently, immunotherapy and targeted therapy have emerged as the standard of HIF-targeting cancer therapeutics. Indirect targeting of the HIF-pathway has shown clinical success in treating solid cancers, with multiple downstream HIF-1α inhibitors developed and FDA approved. For instance, the anti-VEGF monoclonal antibody bevacizumab (Avastin) was first approved in 2004 for the treatment of colorectal cancer in combination with chemotherapy, and has since expanded to the treatment of non-squamous non-small cell lung cancer (NSCLC), ERB2 negative breast cancer, renal cell carcinoma, and glioblastoma [32]. In addition, bevacizumab was the fifth top-selling monoclonal antibody in 2018, emphasizing its clinical benefit [33]. However, there are currently no FDA-approved direct HIF inhibitors to treat solid cancers [34]. A 2011 pilot study suggested topotecan (Hycamtin), an FDA-approved chemotherapeutic agent authorized for certain solid cancers, as an HIF-1α-targeting cancer therapeutic agent because of its ability to inhibit HIF-1α independently from topoisomerase 1 [35,36]. However, the results failed to allow topotecan as a HIF-targeting cancer therapeutic agent because of high toxicity and low specificity [37]. This result highlights the complexity of the HIF pathway and suggests that targeting HIF-1α directly and independently can influence therapeutic efficacy. Additionally, the hypoxic response and the TME are complex. The incorporation of HIF-2α will introduce the spatiotemporal control of the hypoxic response along with novel direct and indirect targets. We propose that the next generation of hypoxia-targeting therapeutic agents will require a dynamic approach to treatment, utilizing both HIF-2α and HIF-1α, mimicking the native hypoxic response and TME. Therefore, considering the role of HIFs in regulating cellular oxygen homeostasis and tumor progression, a comprehensive review of HIF-2α may be necessary to prompt the next generation of hypoxia physiology and pathophysiology research with the end goal of novel therapeutics. In this review, we elucidate the role and regulation of HIF-2α in oxygen homeostasis over the lifetime of the cell in terms of transcription, translation, and protein stability, contrasting HIF-1α when necessary. Then, we review the role of HIF-2α in tumor progression, with emphasis on the spatial and temporal dynamics of the TME. Finally, the consequences of hypoxia on cancer therapy and current therapeutic interventions will be discussed, including our take on the future of hypoxia-mediated research.

## 2. Role of HIF-2α in Development

HIF-2α was first discovered and cloned in 1997 by four individual groups [38,39,40,41]. This discovery came six years after HIF-1α was cloned, revealing the first insights on how cells sense and respond to hypoxia [18,19]. Each group named the newly discovered protein differently, but the accepted naming convention is that the gene, *EPAS1* (endothelial PAS domain protein 1), encodes the protein HIF-2α.

Knockout mouse models of HIF-2α−/−, compared to HIF-1α−/−, were the first to suggest independent roles. Both HIF-1α−/− and HIF-2α−/− mice resulted in premature death at day E10.5, but the cause of death was different for each set. HIF-1α−/− mice exhibited mass cell death and drastic and atypical vascular regression, malformations, and remodeling [15,42,43], while HIF-2α−/− mice died due to bradycardia (failed catecholamine synthesis), respiratory distress syndrome (inadequate alveolar type 2 cell surfactant production), and failed fusion and assembling of primary vasculature [44,45,46]. Later developmental studies using congenic mouse F1 hybrids that carried a null *EPAS1* allele observed hepatosteatosis, cardiac hypertrophy, pancytopenia, metabolic crisis (anion-gap acidosis and altered mitochondrial intermediates), lower body weight, and premature death [47]. Thus, this indicates that both HIF-1α and HIF-2α are essential to oxygen homeostasis, but HIF-2α has a distinct role.

Originally it was suggested that HIF-2α was only expressed in endothelial cells, hence the name *EPAS1*. Now, HIF-2α is known to display spatial expression patterns, being primarily expressed in highly vascularized organs such as the heart, liver, lung, brain, kidney, intestines, pancreas, and uterus [24,25,26]. Within those organs, HIF-2α also exhibits cell-specific expression patterns, with parenchymal expression observed in the intestines and liver; nonparenchymal expression in the kidneys, pancreas, and brain; and uniform distribution observed in the myocardium [26]. Altogether, this contributes to lung maturation [44], catecholamine homeostasis and developmental cardiac function [45], reactive oxygen species (ROS) maintenance and mitochondrial homeostasis [47], vascular remodeling [46], iron homeostasis [48,49], angiogenesis in the retina [50], and is the primary mediator of erythropoiesis [50,51,52,53]. The differential effects that HIF-2α exhibits on the pathology of knockout mice, its diverse roles, and tight spatiotemporal regulation reveals the benefits of HIF-2α in both the development and homeostasis of vertebrates.

### 2.1. Structure of EPAS1

**Figure 1:**
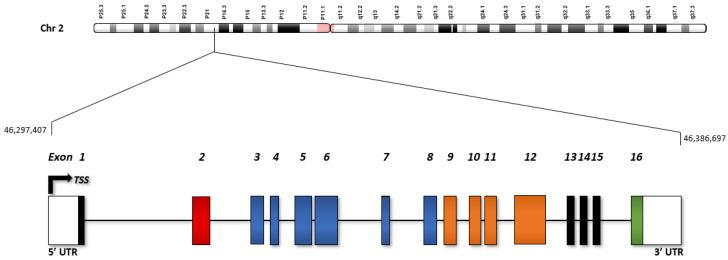
Structure of the *EPAS1* gene on chromosome 2 (Gene ID: 2034).

*EPAS1* is located on chromosome 2, contains 16 exons, and has a promoter region that spans approximately −1988 bp to +100 bp from the transcription start site (TSS) (Figure 1) [54]. Constitutive transcription requires a careful balance of evolutionary conservation and adaptability. In fact, 91% of the *EPAS1* promoter, region −1823 bp to +83 bp from the TSS, is classified as a CpG island (GC content: 61%, O/E ratio: 0.6, length: 1907 bp) [55]. CpG islands are widely associated with the TSS, and overlap with transcriptional regulatory regions, including enhancers, repressors, and promoters [56]. These “CG”-rich regions, especially promoters, are generally protected from methylation by histone acetyltransferases, contributing to genomic evolutionary conservation by minimizing epigenetic regulation [57,58,59]. An intergenic and intragenic CpG island (region −756 bp to +2090 bp from the TSS) was identified in *EPAS1* to be a spot for transcription factor binding and histone acetylation, indicating high levels of gene expression and modularity [60,61,62].

### 2.2. Genetic Variations of EPAS1

Identification of single nucleotide polymorphisms (SNPs), haplotypes, indels, and transcriptional regulators within *EPAS1* indicate how the hypoxic response and oxygen homeostasis are adaptive at the DNA level. Unique haplotypes in *EPAS1* are associated with a high-altitude adaptation in native Tibetans [54,63,64], Tibetan dogs [65], and Himalayan wolves [66], indicating that *EPAS1* is highly subject to positive selection in low oxygen environments. Specifically, an SNP found within the promoter region of *EPAS1* (rs56721780: G>C) is common to the Tibetan population. This mutation decreases the binding of the transcriptional repressor IKAROS family zinc finger 1 (IKZF1) to the *EPAS1* gene, leading to increased expression of HIF-2α. Similarly, an insertion mutation found within the *EPAS1* promoter at the −742 indel is common in Tibetans and provides a binding site for the transcriptional activator specificity protein 1 (Sp1), which also increases the expression of HIF-2α [54]. A genotype comparison between Tibetan and Chinese Han populations revealed three intronic *EPAS1* SNPs (rs13419896, rs4953354, and rs1868092) specific to Tibetans that directly correlated with low hemoglobin concentration [67,68].

### 2.3. HIF-2α Mediates Hypoxia-Induced Translation

Translation is one of the most ATP-consuming processes in cells, especially cap-dependent translation [69]. In an effort to conserve energy and maintain oxygen homeostasis in response to hypoxia, overall cellular protein translation is suppressed up to 93%, requiring alternative hypoxia-induced translation pathways [70]. While HIF-dependent and HIF-independent pathways contribute to this suppression (see review by Chee et al. [71]), HIF-2α can mediate cap-dependent oxygen-independent translation [71,72].

During periods of low oxygen, HIF-2α, RNA-binding motif protein 4 (RBM4), and eukaryotic translation initiation factor 4E type 2 (elF4E2) can form the HIF-2α:RBM4:eIF4E2 complex which binds to RNA hypoxic response elements (rHREs). RBM4 and elF4E2 are typically associated with translational repression, but they switch to translational regulators during periods of low oxygen [73,74]. The HIF-2α:RBM4:eIF4E2 complex is assembled on rHREs, a short ribonucleotide sequence similar to RBM4 binding sites, except for containing a “CCG” motif located in the 3′ untranslated region (UTR) of hypoxia-induced target genes [72]. Formation of the HIF-2α:RBM4:eIF4E2 complex on rHREs enables binding to the 7-methylguanosine 5′ cap on mRNAs, resulting in cap-dependent translation independent of eIF4E. Both epidermal growth factor receptor (*EGFR*) and insulin-like growth factor-1 (*IGF1R*) contain rHREs and are translated by the HIF-2α:RBM4:eIF4E2 complex [75].

### 2.4. Translational Regulation of HIF-2α

RNA binding proteins (RBPs) and microRNAs (miRNAs) are two classes of regulatory molecules that can influence translation rates by binding to the 3′ or 5′ UTR of specific targets. Two RBPs, iron regulatory protein (IRP) 1 and 2, can interact with HIF-2α mRNA, repressing its translation rate [76]. In addition, Zimmer et al. showed that the translational repression of HIF-2α is predominantly regulated by IRP1 [77]. The 5′ UTR of HIF-2α mRNA contains a conserved iron response element (IRE), slowing HIF-2α translation when both oxygen and iron concentrations are low [78]. This iron-mediated feedback mechanism is specific to HIF-2α because it is the prime mediator of erythropoiesis, preventing excessive red blood cell production from disrupting oxygen delivery. Additionally, miR-30a-3p, miR-30c-2-3p, and miR-145 were shown to repress HIF-2α translation by binding to the 3′ UTR [79,80].

### 2.5. Structure of HIF-2α

**Figure 2:**
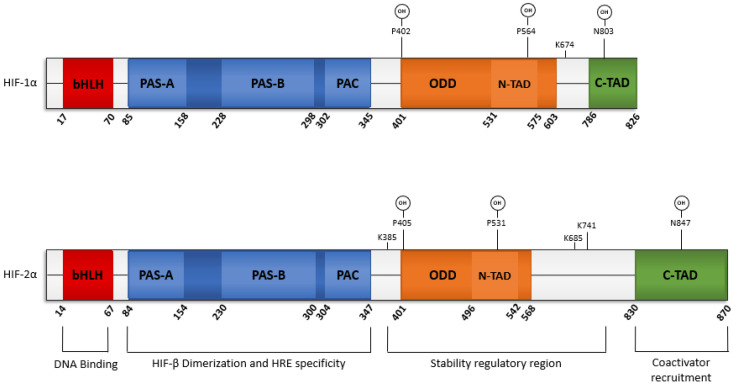
Domain structure of HIF-1α and HIF-2α and their potential function as a transcriptional activator.

HIFs constitute a family of basic helix-loop-helix/PER-ARNT-SIM (bHLH-PAS) heterodimeric transcription factors. The bHLH-PAS family contains highly conserved structural domains that distinguish them as transcriptional regulators, accounting for the high homology of HIF-1α and HIF-2α (Figure 2) [81]. The N-terminal region of the protein includes the most evolutionary conserved motifs, specifically the bHLH domain and PAS domain. The bHLH domain is responsible for DNA binding and contributes to protein dimerization. The PAS domain is composed of PAS-A, PAS-B, and the PAS-associated-C-terminal (PAC), which ensures gene selectivity, heterodimerization specificity, and enables binding of post-translational modifiers [82,83]. In contrast, the C-terminal region is the most variable part of the protein, containing transactivation domains (TADs) and repression domains that give the bHLH-PAS family their diverse functions. HIF-2α contains the evolutionary conserved bHLH-PAS motifs in the N-terminal region along with two TADS: N-terminal TAD (N-TAD) and C-terminal TAD (C-TAD). In addition, HIFs selectively bind to hypoxia response elements (HREs) which have the core DNA sequence 5′-RCGTG-3′, and are located in the promoter regions of genes [84]. Interestingly, HIF-2α was found to bind to a reverse order HRE, sequence 5′-CACGY-3′, located within the promoter region of the membrane-type-1 matrix metalloproteinase gene [85]. To our knowledge there has been no evidence that HIF-1α binds to any reverse order HREs.

### 2.6. PTMs Regulate HIF-2α Stability

**Figure 3:**
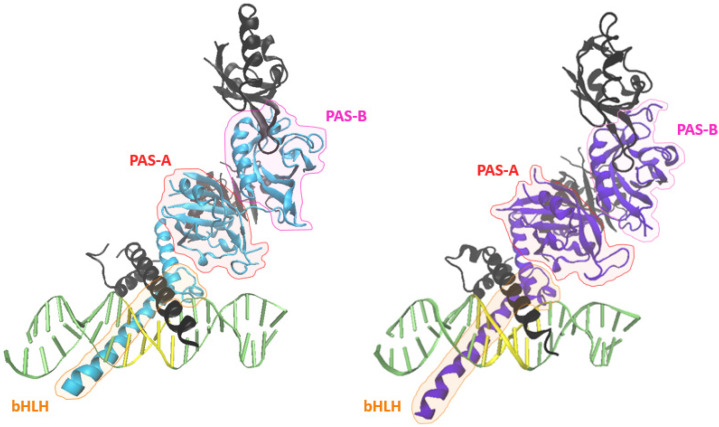
Crystal structure of HIF-1α:HIF-1β (**left**) and HIF-2α:HIF-1β (**right**) heterodimeric complexes bound to a HRE (yellow) (PDB: 4ZPR, 4ZPK) [110].

HIF-α mRNA levels do not correlate with hypoxia-mediated HIF function [86,87,88,89]. The canonical roles of HIF-α are dependent on protein stability, and therefore are primarily regulated by post-translational modifications (PTMs). In fact, the half-life of HIF-1α and HIF-2α is about 5–8 min under normoxic conditions due to PTMs [90,91]. The predominant PTM responsible for the canonical oxygen sensing role is an oxidation reaction, specifically hydroxylation. Hydroxylation of the α-subunit can initiate ubiquitin-mediated proteolysis and block transcriptional coactivator recruitment, leading to the short half-life previously mentioned [92]. Prolyl hydroxylase (PHD) 1-3, and an asparaginyl hydroxylase termed factor inhibiting HIF (FIH), are responsible for HIF-hydroxylation. All four hydroxylases belong to the Fe(II) and 2-oxoglutarate (2OG)-dependent oxygenase superfamily and require oxygen, 2OG, ascorbate, and iron as cofactors [93]. PHD1-3 catalyzes trans-4 hydroxylation of proline residues 405 and 531 in the oxygen degradation domain of HIF-2α (Pro402 and Pro564 in HIF-1α, respectively). Enzyme abundance, dependent on the cell type or cellular oxygen level, primarily determines the catalyzation of the α-subunit by any PHD. PHD2 is considered the principal isoform because of its ubiquitous and basal expression [94,95]. Specifically, PHD2−/− knockout mice died by embryonic day 14.5 while PHD1−/− and PHD3−/− knockout mice developed normally [93,96]. Interestingly, Appelhoff et al. demonstrated that PHD3 is more effective at inhibiting HIF-2α, while PHD2 is more influential over the suppression of HIF-1α [93]. Once hydroxylated, the α-subunit serves as a substrate, binding the von Hippel–Lindau protein (pVHL). Upon binding, pVHL recruits elongin C, elongin B, cullin-2, and ring-box 1 proteins, creating the pVHL complex which together serve as the E3 ubiquitin ligase [97]. The pVHL complex then targets the α-subunit for polyubiquitination, and therefore proteasomal degradation, through recruitment of the E2 ubiquitin-conjugating enzyme [98]. Ubiquitination of HIF-α is efficient for maintaining continuous oxygen sensing and enables a quick response to hypoxia [99]. In normoxic conditions, FIH catalyzes the β-hydroxylation of asparagine residue 847 on HIF-2α (N803 on HIF-1α) to downregulate transactivation [92,100]. This process prevents HIF-α from associating with the transcriptional coactivator E1A binding protein p300/cAMP-response element-binding protein (p300-CBP), inhibiting the transcription of hypoxic-induced genes in normoxia [99,101,102]. Hypoxia stabilizes HIF-α and associates it with p300-CBP and HIF-β to form a transcription factor that binds to HREs (Figure 3). However, despite HIF-1α and HIF-2α having high structural homology and similar oxygen-mediated destabilization, they display different cell-type specific targets and gene specificity though their N-TADs [27,103,104], proximal vs distal promoter binding bias [27,28], stabilization in different oxygen levels [105,106], and durations of hypoxic response [107,108,109].

### 2.7. Spatiotemporal Dynamics of HIF-α

The differences between HIF-1α and HIF-2α indicate distinct spatiotemporal regulation and even coregulation of oxygen homeostasis [111]. Hu C et al. demonstrated that the N-TAD regions of HIF-1α and HIF-2α show different target-gene specificity [112]. Deleting the N-TAD regions in HIF-α abolished this specificity and swapping N-TADs was sufficient to switch target gene expression [104]. A genome-wide mapping study showed that 80% of HIF-2α binding sites resided within distal enhancer regions farther than 2.5 kb from the TSS, compared to 60% of HIF-1α binding sites [28]. Additionally, 425 and 400 high-stringency HIF-2α and HIF-1α binding sites were identified, with some being intronic, intergenic, exonic, and within the 5′ UTR and 3′ UTR. Furthermore, Smythies et al. demonstrated that HIF-1α and HIF-2α have distinct roles by introducing a frameshift mutation through a Cas9 double stranded break, causing a knockout by a premature stop codon in HIF-α target genes [27]. Taylor et al. described that HIF-1α and HIF-2α have different molecular mobilities and sub-nuclear distributions, attributing to the homogeneous nuclear localization of HIF-1α and the ability of HIF-2α to move freely into the nucleus and form speckles [113]. Essentially, this demonstrates that HIF-α behaves independently and does not compete for binding sites throughout the duration nor degree of the hypoxic response [27]. Consequently, this translates into both shared and unique downstream targets. In fact, in endothelial cells, HIF-2α regulated 1454 genes while HIF-1α regulated 701 genes, with 303 overlapping targets [114]. Examples of HIF-2α target genes include: angiopoietin2 (*ang2*), cyclin D1 (*CCND1*), delta-like ligand 4 (*D114*), erythropoietin (*EPO*), peroxisome proliferator-activated receptor alpha (*PPARα*), and periostin (POSTN) [114,115,116]. In comparison, HIF-1α induces phosphoglucomutase 1 (*PGM1*), solute carrier family 2 member 1 (*SLC2A1*), phosphofructokinase (*PFK*), nitric oxide synthase 2 (*NOS2*), carbonic anhydrase 9 (*CA9*), and hexokinase 1 (*HK1*) [52,115,117]. Shared targets include *VEGF*, fibroblast growth factor (*FGF*), transforming growth factor (*TGF*), and angiopoietin-like 4 (*ANGPTL4*) [114]. Altogether, this demonstrates the diverse and complementary role of HIF-α in physiology, and further alludes to its role in tumor progression.

Interestingly, in oxygen concentrations of less than 1%, both HIF-1α and HIF-2α are stabilized, with HIF-1α generally having a higher initial accumulation, and therefore mediating the response to acute hypoxia. However, prolonged hypoxia reverses this, increasing HIF-2α accumulation and decreasing HIF-1α, even when the oxygen concentration increases to around 5%. As a result, HIF-2α is recognized as mediating the response to chronic hypoxia. The shift between acute and chronic hypoxia is generally considered to be 24 h, but it should be noted that this time frame is not always consistent throughout publications, with groups classifying the transition as taking minutes while others consider it to take weeks [118]. Altogether, the spatiotemporal dynamics of HIF-α indicate a clear switch from HIF-1α to HIF-2α in response to prolonged hypoxia [98,119,120].

### 2.8. PTMs Regulate the HIF Switch

Given the importance of PTMs for HIF-α protein stability and the dynamic expression of HIF-α throughout hypoxia, it is unsurprising that PTMs also govern the switch from HIF-1α to HIF-2α. Recently, Zheng et al. identified a positive feedback loop between HIF-1α and HIF-1α anti-sense long non-coding RNA (HIFAL) that would contribute to a higher accumulation of initial expression levels of HIF-1α compared to HIF-2α [121]. Additionally, CBP-p300-associated factor (PCAF), an acetyltransferase that stabilizes the stress-induced p53 pathway, can acetylate HIF-1α at K674, increasing protein levels [122,123]. However, CBP alone can also acetylate HIF-2α within its C-terminus at K385, K685, and K741, deactivating it [124]. Heat shock protein 90 (Hsp90), which increases expression in hypoxia, can stabilize both HIF-1α and HIF-2α by binding to the PAS domain, maintaining high HIF-1α levels and basal HIF-2α levels [125,126,127]. Despite this initial surge in HIF-1α, prolonged hypoxia results in the destabilization of HIF-1α and the increased accumulation of HIF-2α. Receptor-activated kinase C1 (RACK1) competes with Hsp90 to bind to the PAS-A domain of HIF-1α and recruits elongin-C, promoting oxygen-independent proteasomal degradation of HIF-1α, but not HIF-2α [125,126,127]. Similarly, hypoxia-associated factor (HAF), an E3 ubiquitin ligase, gradually increases expression in hypoxia, and selectively degrades HIF-1α but not HIF-2α [128,129]. Sirtuin 1 (SIRT1), a redox sensing NAD-dependent deacetylase, is downregulated in acute hypoxia and upregulated by a redox imbalance, similar to that found in chronic hypoxia [123]. SIRT1 deacetylates HIF-1α at K674, blocking p300 recruitment, leading to decreased expression levels, and deacetylates HIF-2α at K385, K685, and K741, leading to increased expression levels [130]. The transition from acute to chronic hypoxia is also regulated at the transcriptional level by repressor element 1-silencing transcription factor (REST) [131]. HIF-1α drives the transcription of REST, which in turn inhibits the expression of HIF-1α, but not HIF-2α, in a negative feedback loop [132]. Furthermore, Kruppel-like factor 2 (KLF2) is a transcriptional regulator within a zinc finger family subclass that moderates cell differentiation and growth, specifically maintaining gene expression in endothelial cells and regulating pro-inflammatory regulation [133]. KLF2 protein expression is induced during hypoxia within endothelial cells and is downregulated by miR-200b during acute hypoxia [134]. Studies show that KLF2 significantly lessens the accumulation of HIF-1α protein and inhibits the expression of the protein [134,135]. Overexpression of KLF2 can also result in the inhibition of HIF-1α and the genes it targets and selectively promotes HIF-1α degradation. However, like REST, KLF2 has no impact on HIF-2α gene expression or the protein’s stability. Knowing KLF2’s modulating properties, regulating KLF2’s expression allows switching between proteins HIF-1α and HIF-2α during hypoxia. At the translational level, miR-429 is upregulated during hypoxia in some cell types, decreasing HIF-1α mRNA levels [136]. Additional factors that may influence this switch are double minute 2 protein, casein kinase 1δ, and protein kinase D1 [137,138,139].

## 3. Role of HIF-2α in Tumor Progression

Hypoxia arises when the transport of oxygen, nutrients, and waste is reduced below the metabolic demand. Unlike blood cancers that generally do not form masses, solid malignancies’ rapid and uncontrolled proliferation create a mass, forming a TME. Hypoxia develops due to uncontrolled proliferation, the altered metabolism of malignant cells, and aberrant vasculature present in the TME.

### 3.1. The Hypoxic TME

The TME is defined as the dynamic and bi-directional network that surrounds cancer cells and promotes tumor progression. The tumor stroma, the cellular and noncellular components that interact with cancer cells and aid progression, can consist of blood vessels, immune cells, signaling molecules, fibroblasts, and the extracellular matrix. The physical and biochemical interactions within the TME are defining features of cancer progression [140]. Solid tumors exhibit heterogeneity because of inconsistent resources that arise from a rapid proliferation of cancer cells. This intertumoral heterogeneity results in tumor and stroma residing in distinct regions of varying oxygen concentrations or variable hypoxic niches. Unfortunately, solid cancer sites have an approximate detection threshold of 1 cm^3^, which is roughly equivalent to 1 g or 10^9^ cells [141]. New diagnostic methods, including probes, medical imaging, and biomarkers, have been developed to try and reduce this threshold [142,143]. For some cancers, including clear cell renal carcinoma (ccRCC), the initial volume is an even more vital prognosis factor than tumor grade [144]. A tumor volume of 1 cm^3^ equates to a radius of 10,000 μm; comparatively, oxygen diffusion is around 150 μm, and in poorly perfused areas, it can fall to 100 μm [145,146]. Therefore, tumor masses can show hierarchical tissue regions of decreasing oxygen concentration further into the tumor core, resulting in heterogeneous normoxic to necrotic tumor cells and, as a result, differential expression of HIF-1α and HIF-2α. In an attempt to outline the spatiotemporal dynamics of hypoxia within the tumor microenvironment, subtypes have been loosely defined as acute, chronic, or cyclic (also called intermittent) [119]. There are discrepancies involving the temporal categorization of solid tumors, with some groups only examining acute versus chronic [147], while others only compare cyclic and chronic [143,148]. Similarly, there is little consensus regarding which of these subtypes contributes the most to aggressive cancer phenotypes, with separate groups finding that acute [118], chronic [149,150,151,152], or cyclic [153] tumor microenvironments best promote tumor growth. Nonetheless, all subtypes contribute to the co-evolution of cancer progression and TME development, exploiting hypoxia adaptation and promoting non-transformed cell types into pro-tumor stroma phenotypes. The need for a more well-defined hypoxic scale has been identified, perhaps one that strictly defines acute hypoxia as within 24 h.

HIF-2α expression has been detected in many solid cancer sites (Table 1). Roig et al. recently conducted a meta-analysis that included 6052 patients over 18 solid cancer sites to determine if HIF-2α overexpression and clinical outcomes overlapped [154]. The results revealed that HIF-2α levels correlated with poor overall survival, disease-free survival, disease-specific survival, metastasis-free survival, and progression-free survival. The hypoxic TME triggers the overexpression of HIF-2α in both tumor cells and tumor stroma, directly influencing metastasis, angiogenesis, and stemness. Overall, both HIF-1α and HIF-2α are associated with a poor prognosis.

### 3.2. EPAS1 Mutations and Cancer

Somatic mutations, germline mutations, and SNPs in *EPAS1* are associated with tumorigenesis and polycythemia, a rare disease connected to certain malignancies [169]. The ClinVar database contains over 179 mutations within *EPAS1*, while HIF-1α only has 32 mutations identified [170].

Pheochromocytomas and paragangliomas (PPGLs) are rare neuroendocrine-derived catecholamine-secreting malignancies, known for being the most inheritable tumors in humans. In approximately 40% of these malignancies, a mutation of just 1 of 15 PPGL oncogenes is detected [171]. Unsurprisingly, *EPAS1* mutations are one of the most frequent mutations in PPGLs, aligning with its role in catecholamine homeostasis and erythropoiesis. Approximately 40 somatic/mosaic mutations have been identified in PPGL patients (reviewed elsewhere in Toledo et al.) [171]. Specifically, a gain-of-function mutation in exon 12 (c.1589C>T) or exon 9 (c.1121T>A) is responsible for all 13 cases of PPGLs with polycythemia [172,173,174]. Although specific *EPAS1* germline mutations (c.1603A>G, c.1609G>A, c.1609G>T) and somatic mutations (c.1595A>G, c.1586T>C) lead to polycythemia and not tumorigenesis, the onset of polycythemia might itself be a precursor to multiple diseases such as Pacak–Zhuang syndrome, for example [68,173,175,176].

Eight variations in *EPAS1* (c.1084C>T, c.1099C>A, c.1145_1145delT, c.1093C>G, c.1121T>G, c.1137_1137delG, c.1135_1136insT, c.1091_1092insT) were recently detected in 6 out of 80 patients (7.5%) with esophageal squamous cell carcinomas, with the majority of patients carrying these variations experiencing a deregulation of *EPAS1* [177]. These factors directly correlated with tumor location and stage. An SNP within intron 1 (rs13419896) played a role in increasing both the expression and transactivation activity of *EPAS1*/HIF-2α [178]. Patients with non-small-cell lung cancer who had this SNP had a poorer prognosis than patients without this variation.

In addition, mutations in genes associated with oxygen-dependent HIF destabilization create a pseudohypoxic phenotype and lead to further upregulation of HIF-α subunits. An example of this is seen in ccRCC, the most common type of kidney cancer. Histologically, ccRCC is very heterogeneous, and oncogenesis is associated with *VHL* syndrome and heterozygous germline mutations in the *VHL* gene [179]. This *VHL* mutation results in the overexpression of HIF-α subunits. Interestingly, the expression of HIF-2α was an independent predictor of overall survival rate [155]. Furthermore, HIF-1α and HIF-2α display opposite functions, where target genes of HIF-1α correlated with improved survival and decreased tumor growth while HIF-2α target genes correlated with a worse prognosis and increased tumor growth [180]. However, a recent study demonstrated in an autochthonous ccRCC mouse model that HIF-α showed opposite effects, but proposed that both play a crucial role in progression, indicating the importance of a heterogenous TME and HIF-switch regulators [181].

### 3.3. Stroma

There has been increasing interest in the role of HIFs in stroma biology. It is now widely accepted that the TME evolves with tumor progression, giving rise to diverse cell populations with a range of phenotypes in the TME [140,182]. Hypoxic stress has been raised as a common environmental driver of this evolution, and it is therefore of no surprise that HIFs are a focus [182]. Furthermore, the fact that tumor stroma can acquire pro- and/or anti-tumorigenic phenotypes indicates divergent molecular mechanisms [183,184]. The role of HIF-2α in stroma biology was first observed in tumor-associated macrophages (TAMs), one of the most abundant cell types in the TME [185]. Talks et al. observed strong immunostaining of HIF-2α in TAMs associated with several solid tumor types, including lung, liver, and breast, in addition to differentiated U937 cells [186]. Overexpression of HIF-2α, compared to HIF-1α, was confirmed in TAMs found within primary invasive breast carcinomas, and was associated with a higher tumor grade [187]. Thus, to understand the role of HIF-α in macrophage polarization, Takeda et al. measured HIF-2α expressed after Th1 or Th2 cytokine administration in polarized macrophages [116]. HIF-1α and HIF-2α showed antagonistic functions, with HIF-2α induction by Th2 cytokines in M2 macrophages. HIF-2α and HIF-1α correlated with arginase 1 genes and inducible nitric oxide (NO) synthase genes, respectively, which hints that HIF-2α may regulate the cellular oxidative state [188]. Finally, Casazza et al. suggested that HIF-2α plays a role in trapping TAMs in the hypoxic TME by activating nuclear factor-κB (NF-κB) and repress neuropilin 1 (Nrp1) [182].

Given HIF-2α was first thought to be solely expressed in endothelial cells (ECs), HIF-2α may contribute to tumor progression by regulating ECs. Indeed, Skuli et al. demonstrated that knocking out HIF-2α in ECs reduced tumor vascularization and growth in Lewis lung carcinomas (LLC) and B16F1 melanoma cell line xenografts [189]. The predominance of HIF-2α compared to HIF-1α in ECs was confirmed with PHD2 knockdown in ECs, where a 50% reduction in PHD2 resulted in higher HIF-2α expression and was shown to bind to the promoter region of *Flt1* and *VE-cadherin*, presumably to improve vascular perfusion [190]. Interestingly, tumor growth did not change, and metastasis was reduced when comparing PHD2^+/−^ ECs and wild-type LLC in Panc02 mouse models. While this does reassure HIF-2α’s role in vessel remodeling and integrity, it is contradictory to a pro-tumorigenic role of HIF-2α. However, it may give insight into the feedback mechanisms used to fuel tumor progression in the TME. For example, extracellular superoxide dismutase (SOD3), an antioxidant enzyme that catalyzes extracellular superoxide free radicals, is downregulated in many solid cancer types, increasing oxidative stress, a cancer hallmark [191,192,193]. Re-expression of SOD3 to the TME inhibited PHD activity, and therefore induced promoter-driven transcription of vascular endothelial (VE) cadherin by HIF-2α [194]. As a result, vessel hyperpermeability improved and increased chemotherapeutic delivery. Only HIF-2α overexpression led to an increase in vascular endothelial cadherin expression, and the authors suspect that this is because HIF-1α had a more rapid decay. In addition, SOD3 overexpression in ECs increased the transmigration of T-lymphocytes by upregulating laminin-α4 (LAMA4) in an HIF-2α-dependent manner [195]. Altogether, this indicates a unique role of HIF-2α in stroma ECs, and emphasizes the dynamic role the TME plays in tumor progression.

The role of HIF-α in immune exclusion, and specifically in T-lymphocytes, has been explored in many studies [196]. Most notably, HIF-2α is essential for regulatory T (Treg)-cell development in mice. Knockout of HIF-2α in Foxp3-specific cells resulted in the inability to suppress colitis induced by effector T-cells and a resistance to tumor growth, indicating the potential to target HIF-2α in the TME to control immune tolerance [197]. Singh et al. found that miR-15b/16 regulate the expression of HIF-1α and HIF-2α encoding *EPAS1* in helper T-cells. This suppressed the differentiation of induced Tregs and promoted the expression of IL-9 in Th9 cells.

### 3.4. Metastasis

The development of a secondary malignant site, termed metastasis, typically correlates with advanced stage cancers and a shorter overall survival [198]. As first described by the ‘seed and soil’ hypothesis in 1989, the bi-directional communication between cancer cells and the TME promotes metastasis [199]. In fact, it has been suggested that hypoxia contributes to the regulation of every step of metastasis, and therefore positions HIFs as metastatic ‘master regulators’ [200]. Indeed, HIF-2α has been identified to interact with 70 proteins in 501mel melanoma cells, most notably SOX10 and AP2a, alluding to its key role in cancer development [201]. While metastasis is a complicated and multi-step process, it is clear that the adaptation to oxidative stress is directly associated with transdifferentiation and metastasis in cancers [202,203,204,205].

The developmental role of HIF-2α in ROS and mitochondrial homeostasis suggests that it may play a role in adapting to the oxidative stress caused by hyperproliferation. Conditions of high oxidative stress are cytotoxic to cells and the upregulation of antioxidants is required to maintain redox balance. A typical antioxidant that regulates oxidative stress and maintains redox balance in the reduced state is thioredoxin (TXN). Notably, TXN is shown to be induced by hypoxia, specifically HIF-1α, in hepatocellular carcinoma (HepG2) cells [206]. The exploitation of antioxidants, like TXN, is common in many cancer cells. Increased expression of TXN in hepatocellular carcinoma cells correlated with increased HIF-2α stability by PTMs [166]. The dissociation of SENP1 from TXN leads to SUMOylation of SIRT1, enabling SIRT1 to deacetylate HIF-2α, thus increasing its expression. SUMOylated SIRT1 regulates HIF-1α in an opposite manner, demonstrating the HIF switch in vitro. Upregulated TXN and HIF-2α both in vivo and in vitro were also associated with epithelial-mesenchymal transition (EMT) and metastasis [207].

### 3.5. Angiogenesis

The expression of HIF-2α in highly vascularized cells and its role in oxygen homeostasis, specifically in development and erythropoiesis, suggests that HIF-2α may have evolved to ensure recruitment, remodeling, and maturation of primitive vasculature, even in regions with oxygen concentrations higher than anoxic (>5%). This expression would ensure that impaired vasculature, such as during embryonic development and in the wound healing process, could signal a need for angiogenesis or repair. Moreover, similarities between the wound healing process and TME dynamics resulted in the characterization of cancer as “a wound that will not heal” [208,209]. Indeed, Yamashita et al. demonstrated that HIF-2α knockdown mice transplanted with melanomas had significantly reduced tumor size and fewer large vessels [210]. This result occurred because of the reduced ephrin-A1 expression on vascular endothelial cells, decreasing binding of HIF-2α, but not HIF-1α, to an HRE in the promoter of ephrin-A1. Ephrin-A1 is both a soluble and membrane-bound ligand that binds to its cognate, ephrin type-A receptor 2 (EphA2), and influences cell behavior, regulating cell adhesion and cytoskeleton remodeling during embryogenesis and inflammation [211]. Specifically, in pulmonary vascular endothelial injuries, Ephrin-A1/EphA2 is upregulated, increasing monolayer permeability, and potentially enabling entry of inflammatory cells and fluid [212]. Tumor necrosis factor-alpha (TNF-α), a typical cytokine in both the TME and the early wound healing process, upregulates the expression of ephrin-A1 [213,214]. Upregulation of EphA2 by hypoxia has been noted in multiple cancers and corresponds with the upregulation of the NF-κB pathway, a pathway that links cancer and chronic inflammation [215,216,217,218]. Initial disruption to the endothelial barrier followed by repair and stabilization is a crucial feature of tumor angiogenesis linking the ephrin/Eph pathway as angiogenic mediators [219,220]. Corroborating with previous results, the loss of HIF-2α in endothelial cells indicated impaired tumor growth, increased vessel permeability, and reduced the expression of ang2, D114/Notch signaling, and various cell adhesion molecules [117]. Altogether, this indicates that HIF-2α plays a role in angiogenesis by controlling vascular morphogenesis.

### 3.6. Stemness

Cancer stem cells (CSCs) constitute a small population of intratumoral cells that hold a stem cell-like phenotype (“stemness”). Aggressive environments, heterogeneity, and the TME all fuel the development of CSCs [221,222]. Cancer adaption to intertumoral oxygen gradients created by hypoxia leads to increased heterogeneity and plasticity, common features of an aggressive phenotype [5]. Therefore, CSCs typically reside in anatomically distinct “niches” within the TME; these regions correlate with low oxygen and limited immune evasion. The spatiotemporal regulation of HIF-2α suggests HIF-2α plays a role in mediating stemness. Indeed, Seidel et al. proposed that CSCs are maintained in hypoxic niches, and HIF-2α, but not HIF-1α, upregulates stem-cell surface markers, including CD133 [163]. In melanoma cells, HIF-2α was shown to upregulate miR-363-3p, a miRNA associated with proliferation and invasion in malignant cells and healthy endothelial cells [161,223]. miR-363-3p was shown to directly bind to the 3′ UTR of p21, a controller of the cell cycle, and inhibit its function. Inhibition of p21 causes increased levels of CD133, Jarid1B, and Nanog, all markers of a stem cell-like phenotype [161]. Additionally, HIF-2α was shown to directly bind to the promoter of POU class 5 homeobox 1 (*POU5F1)*, commonly referred to as Oct4, a potent regulator of stem cell pluripotency [224]. In non-small-cell lung cancer (NSCLC) cells, HIF-2α not only promotes the expression of the FOXP3 protein but also binds directly to it, promoting its oncogenic role. HIF-2α overexpression was also shown to induce the expression of nuclear enriched abundant transcript 1 (NEAT1). The NEAT1 and FOXP3 proteins are believed to activate the Wnt/β-catenin signaling pathway, inducing the epithelial-mesenchymal transition in lung cancer cells, and thereby increasing their stemness [225,226]. The role of HIF-2α overexpression in these pathways and its correlation to a poor prognosis in NSCLC patients exemplifies the need for HIF-2α targeting therapies for the disease [227].

## 4. Hypoxia and Cancer Therapy

The impact of hypoxia on tumor progression and therapeutic resistance has established the hypoxic TME as a promising strategy to improve the efficacy of solid cancer therapeutics. Hypoxia is a hallmark of solid tumors and is used for tumor imaging, detection, and prognosis. Hypoxia strongly correlates with a poor cancer prognosis, disease relapse, and acts as a strong obstacle to radio and chemotherapies. Additionally, over 20 common cancer drugs have been shown to be less effective in hypoxic tumors [31]. As previously mentioned, there is a strong significant negative association between HIF-2α expression and survival endpoints [154]. In normoxia, radiation therapy and photodynamic therapy are effective at killing cancerous cells by generating ROS; however, because of the lack of oxygen in hypoxic conditions, these therapies have become less effective at killing tumor cells [228,229]. Specifically, well-oxygenated cells respond to radiotherapy 2.5–3 times better than hypoxic cells, and optimal radiosensitivity is achieved at a partial pressure of oxygen above 20 mmHg [230]. Additionally, large fractions of hypoxic cells found in tumors not only promote a CSC phenotype, but also prevent chemotherapeutics from accumulating at functional concentrations, both of which contribute to therapeutic resistance [231].

Several strategies to mitigate the detrimental effects of hypoxia on cancer therapies have occurred over recent years. The use of hypoxia-activated prodrugs (HAPs), which exclusively activate in low oxygen environments, has shown some promise in the pre-clinical and clinical development stages. For example, TH-302 or evofosfamide, is a promising hypoxia-activated drug in Phase 1 and 2 trials, but has been limited in Phase 3 trials so far due to limited efficacy, as reviewed in [228]. It is hypothesized that poor screening for patient tumor hypoxia levels may be the cause for these limited results [232]. In addition, EO9, also known as apaziquone, is reduced to hydroquinone in the presence of oxygen, but in hypoxic conditions is reduced to semiquinone, which is more toxic to the tumor. EO9 had limited efficacy in Phase 1 and 2 trials, as hydroquinone and semiquinone suffer from instability due to a short half-life, but showed promise for a localized treatment via intravesical instillation for superficial bladder cancers. PR-104 has shown advantages in leukemias by “decreased tumor burden and increased survival” in Phase 1/2 trials, but has shown toxicity and sparse responses in solid tumors. AKR-1C3 can be used as a biomarker to predict the PR-104 response, as it causes the activation of PR-104 independent of tissue oxygenation, resulting in toxicity [233]. CP-506 is a new hypoxia-activated prodrug based on PR-104, but is more resistant to AKR-1C3 activation and is orally bioavailable. In vivo, CP-506 showed strong selectivity for hypoxic cells in tumor xenograft models and is currently in a Phase 1/2 trial [234]. However, no HAPs have yet received FDA approval [235,236]. Due to hypoxia’s role in reducing radiotherapy efficacy, some groups rely on imaging local oxygen partial pressures to determine their radiation dosage painting [237]. Dosage optimization can likely improve outcomes; however, this approach must be tested in a clinical setting to verify the model, and this strategy does not address the underlying issues of the hypoxic environment. Other therapies for reducing hypoxia involve altering the plasma oxygen supply of patients, either by having them breathe hyperbaric oxygen (HBO) or supplemental oxygen. When used in combination with radiotherapy, HBO has been shown to increase patient survival. Reducing hypoxia by increasing blood oxygen levels has been shown to reduce adenosine concentration in the TME, increasing the local activity of natural killer cells and T-cells [31]. It should be noted that HBO treatment requires a specialized facility and 24/7 occupation of the patient during treatment to see its full benefits. Lastly, another hypoxia control strategy is starvation therapy via glucose oxidase in liposomes to deplete the glucose and oxygen supply in the tumor and produce toxic hydrogen peroxide [238]. This method magnifies the hypoxia of the region, allowing for better targeting of hypoxia-activated prodrugs, but retains the radiotherapy disadvantages. Conversely, oxygen therapeutics based on liquid fluorocarbons may be used to increase the oxygen carrying capacity of the blood to reverse hypoxia [31].

Various HIF-1α indirect inhibitors have been developed, with Rapamycin, Cetuximab, Romidepsin, and Vorinostat receiving FDA approval for the treatment of a variety of cancer types; however, as previously mentioned, no direct HIF-1α inhibitors are currently on the market [239,240]. Ma et al. recently compiled a comprehensive review of drugs currently under investigation which indirectly inhibit HIF-1α activity, either by suppressing signaling pathways which upregulate its concentration, promoting its degradation, or by inhibiting its transcription and translation [241]. While targeting HIF-1α has shown some success, it fails to address the oncogenic biochemical pathways involved in tumor progression that are reliant on HIF-2α, as previously discussed. As a result, three small-molecule agonists that specifically target HIF-2α, named PT2385, PT2399, and PT2977, have been developed. These allosteric inhibitors block the dimerization of HIF-2α with HIF-1β. Both drugs have proved efficacious in pre-clinical models of ccRCC and kidney cancer, respectively [242,243]. Encouragingly, a Phase 1 dose-escalation trial of patients with advanced ccRCC treated with PT2399 suggested that the small-molecule inhibitor was tolerated favorably [244]. Similarly, in a Phase 1 trial, PT2385 demonstrated a synergistic inhibitory effect on tumor growth when used as a combination therapy with the anti-PD-1 antibody [245]. However, PT2385 was shown to inhibit HIF-2α activity in non-tumor patients, as shown by a reduction in erythropoietin. This reduction resulted in a functional reduction in red blood cell precursors. Furthermore, tumors may develop resistance to both PT2385 and PT2399 after prolonged treatment via a G323E gate keeper mutation in the HIF-2α gene [246,247]. Nevertheless, these first-of-their-kind drugs highlight the potential for selectively targeting HIF-2α in ccRCC and, theoretically, a broad range of cancers.

Expression of immune checkpoints such as programed death ligand 1 (PD-L1) and cytotoxic T-lymphocyte-associated antigen 4 are associated with poor prognosis and are often upregulated in malignant tumors. Immune checkpoint inhibitors are a promising form of immunotherapy which block the immune suppressing checkpoints displayed by cancers, which allows them to evade the immune response [248]. The success of these therapies indicates the importance of reducing immune checkpoints in cancerous cells. Hypoxia has been identified as a factor in the upregulation of the immune checkpoint PD-L1 in myeloid-derived suppressor cells. In ccRCC cells, the immune checkpoint PD-L1 and HIF-2α expression are correlated, and targeting HIF-2α results in a decrease in PD-L1 protein and mRNA expression levels [249]. As a complex, HIF-1β and either HIF-1α or HIF-2α induce the expression of a variety of immune checkpoint genes such as PD-L1, CD47, CD137, CD73, and CD70 in hypoxic ccRCC cells [250,251]. The effect that HIF-2α has on these checkpoints emphasizes its potential as a therapeutic target, particularly when used in combination with checkpoint inhibitors. In a Phase 1 trial, the previously discussed small-molecule agonist, PT2385, demonstrated a synergistic inhibitory effect on tumor growth when used as a combination therapy with the anti-PD-1 antibody [245]

Most other HIF inhibitors being investigated act either exclusively on HIF-1α, or on both HIF-1α and HIF-2α. One notable drug that targets HIF-1α and HIF-2α is the histone deacetylase (HDAC) pan inhibitor Vorinostat (Suberoylanilide Hydroxamic acid/SAHA), which the FDA approved for the treatment of cutaneous T-cell lymphomas. There are many theories about the multiple mechanisms of the inhibitory function of this drug. One such theory is that this drug may inhibit stabilization of both HIF-1α and HIF-2α through the acetylation of Hsp90 [252]. Vorinostat can also block the nuclear translocation of HIF-1α and HIF-2α by inhibiting its interaction with Importin [243]. While showing some promise, current therapies are limited by the complexity of the HIF pathway, and a new wave of approaches is required to combat a hypoxic TME.

## 5. The HIF-α Debate

While our understanding of the hypoxic response has grown tremendously in the 10 years since “*HIF1α and HIF2α: Sibling rivalry in hypoxic tumour growth and progression*” was published, the role of HIF-α in tumor progression remains controversial [17]. It is important to note that there are studies that report *EPAS1*/HIF-2α as a tumor suppressor [181,253,254,255,256]. In addition, some studies report HIF-1α as a tumor suppressor [257,258,259,260]. The landmark review by Keith et al. was the first to expound the overlapping and opposing roles of HIF-α in tumor progression [17]. We have reviewed many of the factors that contribute to these divergent roles, including PTMs, cell type, and degree and duration of hypoxia. For example, as previously mentioned, HIF-2α expression in TAMs has been linked to an M2 phenotype [116] and poor prognosis [261], and expression is correlated with tumor grade [186,187]. However, Cowman et al. recently found that TAMs in ccRCC tissue samples primarily expressed HIF-1α, not HIF-2α, and HIF-1α expression significantly correlated with tumor stage [262]. In addition, they found HIF-1α co-localized with CD137, an M2 marker. This recently discovered correlation between HIF-1α expressing TAMs and ccRCC is noteworthy given the evidence of HIF-2α’s role as the predominant driver in ccRCC, and may inadvertently demonstrate the context-dependent roles that HIF-α plays in the stroma to counteract hypoxic stress and drive tumor progression. Furthermore, in a E0771 breast cancer allograft model, HIF-2α deletion in myeloid cells increased tumor growth [263]. This revealed that in breast tumors, HIF-2α expression in TAMs had a tumor-suppressive role by expressing Spint1 (serine protease inhibitor, Kunitz type-1). Recently, single-cell RNA sequencing revealed that TAMs do not exhibit a defined M1 or M2 polarization, as was also seen in Cowman et al., thus further indicating the importance of environmental factors [262]. Nonetheless, HIF-α clearly displays spatial and temporal topography. Despite Keith et al. suggesting these dynamics and proposing that the hypoxic response is balanced by the antagonistic roles of HIF-1α and HIF2α, HIF-2α is still overlooked [17]. At the time of this publication, the term “HIF-1” appeared 7x more than the term “HIF-2” within publications when searched on Google Scholar over the past 10 years (62,900:9070). In addition, the term “HIF-1” was referenced within the title of a publication 10× more than “HIF-2” (2270:224). We argue that physiologically, hypoxia is a dynamic process, but clinically, the role of HIFs is still primarily viewed as players in a static system resulting in limited clinical success.

Although HIF-1α and HIF-2α display complementary interactions, they have distinct roles and are involved in a variety of independent regulatory pathways. Given the spatiotemporal regulation of HIFs, the isoforms may only be expressed at critical points in tumor progression; for example, HIF-1α primarily mediates angiogenesis, while HIF-2α mediates vascular integrity. We postulate that the nature of the tumor and the TME can drive these differential results. For instance, the presence or absence of an oncogene can influence the roles of HIF. HIF-1α and HIF-2α differ in how they regulate the highly oncogenic pathway, MYC, which is seen to be upregulated or downregulated in cancers by up to 70% [264,265]. Generally, HIF-1α disrupts the MYC pathway by transcriptionally displacing MYC binding [266], promoting MYC proteasome degradation [267,268], inducing the expression of MAX antagonists such as MXI1 [267], and competitively binding to MAX and Sp1 [266,269]. This leads to the expression of p21, G1-phase arrest, and the reduction of genes involved in DNA repair and mitochondrial biogenesis. Conversely, HIF-2α can enhance MYC activity by stabilizing and promoting the MAX/MYC complex [138,267]. In addition, the overexpression of HIF-2α can promote Sp1 activity, and therefore IL-8 expression, by MYC [270]. Leading to S-phase entry, genomic stability is preserved through the expression of DNA repair proteins and resistance to replicative stress. However, overexpression of MYC can overcome HIF-1α inhibition, with studies showing HIF-1α can induce specific MYC target genes [271,272]. This can explain the antitumorigenic effects of HIF-1α in cancers with low MYC oncogenic dependency and demonstrates the complexity of HIF-α in highly MYC oncogenic cancers, suggesting that solely inhibiting HIF-1α may result in preliminary attenuation, but have no impact on overall tumor burden [273]. Shih et al. [274] demonstrated that HIF-2α was critical for tumor re-proliferation, i.e., the switch from tumor dormancy to proliferation, in epithelial ovarian cancer cells. In addition, a comparison of the acute hypoxic response while HIF-2α mediates the chronic response then initial proliferation and subsequent reoxygenation due to rapid proliferation would be predominantly controlled by HIF-1α.

Considering it is estimated that over 40% of cancers overexpress MYC, it may be beneficial to target HIF-1α and MYC using overlapping pathways and direct or indirect HIF-2α targets [265]. It should also be noted that similar HIF-1α and HIF-2α dynamics are seen in other critical pathways such as p53 and mTOR, introducing other potential targets which can be exploited by therapeutic interventions [17,275]. Furthermore, genomic abnormalities can influence HIF-α function. A 14q focal deletion is a common abnormality in ccRc; specifically, the deletion encompasses the *HIF-1α* locus and contributes to a loss of function mutation of the *HIF-1α* gene, which is otherwise classified as a tumor suppressor [260].

**Figure 4:**
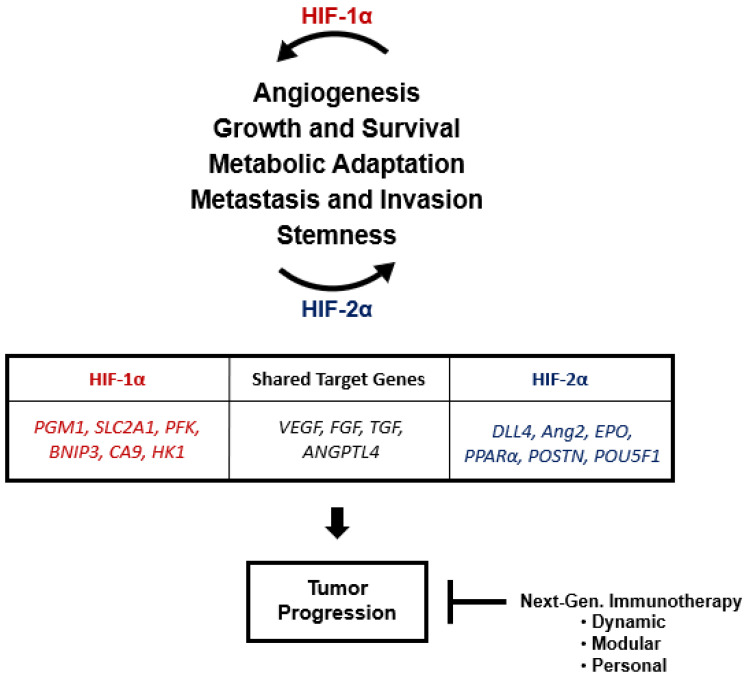
Systematic representation of HIF-1α and HIF-2α on tumor progression. HIF-2α regulates 1454 downstream genes while HIF-1α regulates 701 genes, with a combined 303 overlapping targets [114].

We suggest an alternative perspective to explicate the role of HIF-1α and HIF-2α in tumor progression (Figure 4). We propose that HIF-1α and HIF-2α are continuously optimized and balanced until the ‘end stage’ of tumorigenesis, with varied HIF-α requirements based on location, TME, and stage. For example, Mazumdar et al. demonstrated that the deletion of HIF-2α in a Kras^G12D^-driven murine NSCLC model resulted in tumor growth, whereas HIF-1α deletion did not affect growth. Interestingly, the overexpression of HIF-2α led to angiogenesis and EMT transition [254]. It was concluded that reducing HIF-2α below a threshold resulted in deletion of the tumor suppressor *SCGB3a1,* but overexpression increased *VEGF*, *VEGFR2*, and *snail* [254]. Altogether, this alternative perspective can elucidate unique and opposing HIF-α activities in tumor progression.

## 6. Future Perspectives

The impact of hypoxia on tumor progression and therapeutic resistance has established the hypoxic TME as a promising new strategy to improve the efficacy of solid cancer therapeutics. Immunotherapy, specifically adoptive cell therapy (ACT), is a dynamic and promising target for future generations of solid cancer therapies. Unlike monoclonal antibodies and small molecules, ACT allows the patient’s own immune cells to effectively target, terminate, and persist cancer. The modularity of ACT provides a dynamic and personalized system that can be adapted to include the most recent advances and findings in the field. For example, multiple hypoxia-sensing CAR T-cells have been developed. Juillerat et al. was the first to fuse the HIF-1α ODD to the CAR scaffold, termed HIF-CAR, and demonstrate a CAR T-cell only responsive in hypoxic environments [276]. Recently, two other hypoxia-sensing CARs, termed HiCAR and HypoxiCAR, have been developed, and both utilize the HIF-1α ODD [277,278]. In addition, HiCAR and HypoxiCAR incorporate single or consecutive HREs upstream of their promoter to induce CAR expression in hypoxic conditions, leading to dual hypoxia sensing, and demonstrating the versatility and potential for immunotherapy. Similarly, oxygen-sensing nanoparticles offer an alternative delivery method for existing cancer therapies, and can be used in combination with CARs to target the TME [279].

At the forefront of many developing cancer therapies is CRISPR/Cas9-mediated gene editing. While CRISPR-mediated *HIF-1α* knockout has shown promise in in vitro and in vivo mouse liver cancer models, to our knowledge, currently, no therapies targeting HIF-2α are in development. The modularity of CRISPR technology would likely allow for this system to be easily adapted to target HIF-2α in cancer, however, further development of delivery methods beyond the implantation of lentivirus transfected with CRISPR/Cas9 utilized in this study are required for clinical applications [172]. In addition, epigenetic modifications have shown potential in pre-clinical and clinical trials [244]. A high throughput screen for HIF-2α expression that utilizes CRISPR-Cas9 to target chromatin regulators has been developed to better investigate the epigenetic regulation of the protein. This study found that there are no individual factors that are essential for HIF-2α expression in the ccRCC cells that they investigated, indicating that targeting multiple chromatin factors would likely be required to efficiently regulate HIF-2α [252].

## 7. Conclusions

In the present review, we highlighted the role and regulation of HIF-2α in both physiology and tumor progression, emphasizing the independent and coregulatory dynamics of HIF-2α compared to HIF-1α. We also established the similarities between the spatiotemporal dynamics of the TME and the HIF pathway. However, we also indicated and implied that HIF-1α and HIF-2α are diverse by nature, and proposed an alternative perspective to elucidate the role of HIF-α dynamics in tumor progression; that cancer optimizes the HIF pathway for tumor progression through balancing of the α-subunit. The observation that HIF-α is highly dynamic and spatiotemporally regulated suggests that therapeutic interventions may be challenging, requiring the next generation of solid cancer therapies to be both innovative and dynamic.

## Figures and Tables

**Table 1:** Summary of the overexpression of HIF-2α in multiple cancer types with HIF-1α as a comparison.

Cancer Type	Prognosis	Comparison Made to HIF-1α	Evidence of HIF-1α Involvement	Method(s)	Reference
Neuroblastoma	Advanced clinical stage	No	N/A	Western blot, RT-PCR	[80]
	Angiogenesis, aggressive phenotype, growth	Yes	Transiently expressed	Western blot, qPCR	[105]
	Aggressive phenotype, metastasis	Yes	Transiently expressed	Western blot, RT-qPCR	[106]
	Aggressive phenotype	Yes	Transiently expressed	Western blot, qPCR	[109]
	Stemness	Yes	Transiently expressed	Western blot, RT-qPCR	[89]
Clear cell renal carcinoma	Poor overall survival	Yes	Lower Fuhrman grade	Immunohistochemistry	[155]
	Oxidative phenotype	Yes	Basal expression, decreased growth	Immunohistochemistry	[156]
	Cell cycle progression	No	N/A	Western blot	[157]
Arsenite- transformed liver cancer	Epithelial-mesenchymal transition, stemness	No	N/A	Western blot	[158]
Breast cancer	Worse disease-specific survival (HER2+)	Yes	Independent normal expression	Western blot, RT-PCR, immunohistochemistry	[159]
	Epithelial-mesenchymal transition, invasion	Yes	Independent normal expression	Western blot, qPCR	[160]
Melanoma	Stemness	Yes	Independent overexpression	Western blot, siRNA,	[161]
Glioblastoma	Increasing grade, mortality	No	N/A	Immunohistochemistry	[162]
	Stemness	Yes	Independent overexpression	Western blot, immunochemistry	[163]
Non-small-cell lung cancer	Mesothelial- mesenchymal transition	No	N/A	Western blot, shRNA	[164]
Lung adenocarcinoma	Growth, resistance	No	N/A	qt-PCR, shRNA	[165]
Hepatocellular carcinoma	Metastasis	Yes	Transiently expressed	Western blot, shRNA	[166]
Colon cancer	Resistance	Yes	Co-expressed	Western blot, siRNA	[167]
Cancer Stem Cells	Stemness, self-renewal	No	N/A	qPCR, siRNA, ELISA	[168]

Abbreviations: RT-PCR: reverse transcription-polymerase chain reaction, qPCR: quantitative polymerase chain reaction, siRNA: small interfering RNA, shRNA: short hairpin RNA, ELISA: enzyme-linked immunosorbent assay.
